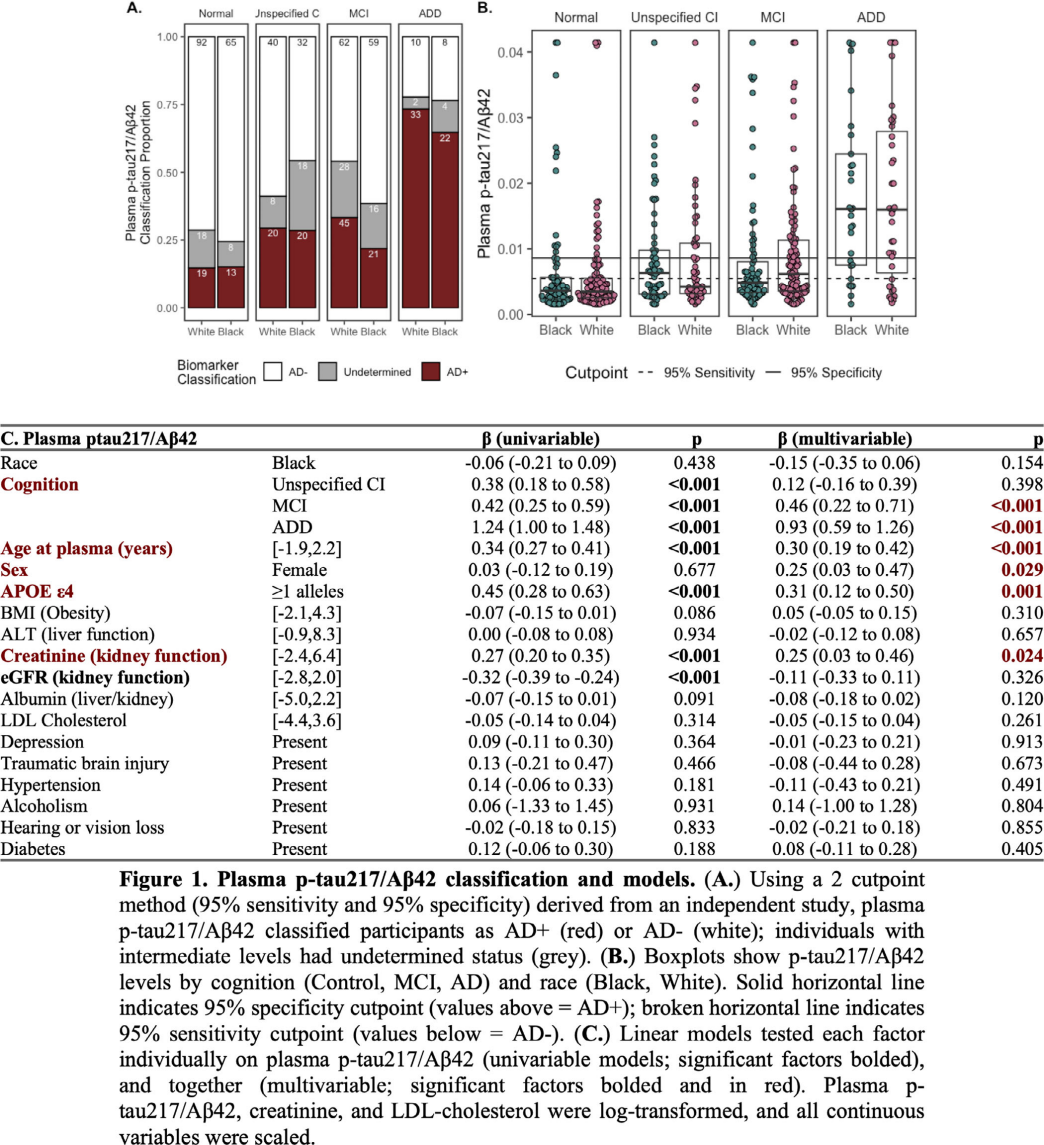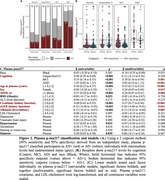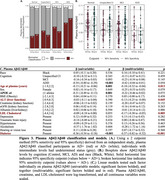# Electronic health records to test influence of race and multimorbidity on plasma biomarkers

**DOI:** 10.1002/alz70856_097254

**Published:** 2025-12-24

**Authors:** Katheryn A. Q. Cousins, Rory Boyle, Colleen Morse, Anurag Verma, Marina Serper, Nadia Dehghani, Corey T. McMillan, Leslie M. Shaw, David A. Wolk, Penn Medicine Biobank

**Affiliations:** ^1^ Department of Neurology, University of Pennsylvania, Philadelphia, PA, USA; ^2^ Department of Neurology, University of Pennsylvania Perelman School of Medicine, Philadelphia, PA, USA; ^3^ University of Pennsylvania, Philadelphia, PA, USA

## Abstract

**Background:**

Plasma biomarkers of Alzheimer's disease (AD) pathology are frequently tested in research settings, limiting generalizability of findings. Using electronic health records, we evaluated plasma biomarkers – phosphorylated tau 217 (*p*‐tau217), β‐amyloid 1‐42/1‐40 (Aβ42/Aβ40) and *p*‐tau217/Aβ42 – in a real‐world, racially diverse data set with multimorbidities.

**Methods:**

Participants (*n* = 683; 298 Black/African American, 385 White) were selected from the University of Pennsylvania Medicine Biobank. International Classification of Disease (ICD) codes determined AD dementia (ADD; *n* = 79), mild‐cognitive impairment (MCI; *n* = 239), unspecified/non‐AD cognitive impairment (CI; *n* = 143) and cognitively normal cases (*n* = 222). ICD codes determined histories of diabetes, depression, traumatic brain injury, hypertension, alcoholism, and hearing/visual loss, and body mass index (BMI). Blood was analyzed for APOE ε4, ALT, creatinine, eGFR, albumin, and LDL‐cholesterol. Plasma analytes were assayed using Fujirebio Lumipulse; previously established cutpoints determined AD status: “AD+”, “AD‐”, or “Undetermined”. Multivariable models tested how morbidities, age, sex, and race related to plasma levels.

**Results:**

Plasma *p*‐tau217/Aβ42 (Figure 1) was increased in MCI (β=0.46, *p* <0.001) and ADD compared to Normal cognition (β=0.93, *p* <0.001); *p*‐tau217/Aβ42 increased with age (β=0.30, *p* <0.001), female sex (β=0.25, *p* = 0.029), APOE ε4 (β=0.31, *p* = 0.001), and creatinine (β=0.25, *p* = 0.024), with no difference by race (*p* = 0.15)

Plasma *p*‐tau217 (Figure 2) was increased in MCI (β=0.38, *p* = 0.001), ADD (β=0.83, *p* <0.001), with age (β=0.23, *p* <0.001), female sex (β=0.24, *p* = 0.015), APOE ε4 (β=0.24, *p* = 0.006), higher creatinine (β=0.37, *p* <0.001), lower eGFR (β=‐0.20, *p* = 0.041), and lower albumin (β=‐0.13, *p* = 0.003); difference by race was not significant (β=‐0.18, *p* = 0.053).

Plasma Aβ42/Aβ40 (Figure 3) decreased with age (β=‐0.18, *p* = 0.013), higher ALT (β=‐0.13, *p* = 0.045), higher LDL‐cholesterol (β=‐0.17, *p* = 0.005), and diabetes (β=‐0.57, *p* <0.001), with no difference by race (*p* = 0.221).

Proportions tests confirmed similar biomarker positivity by race (all *p* ≥0.19). Plasma *p*‐tau217/Aβ42 classified the most cases (AD+/‐; Figures 1‐3), with significantly fewer undetermined cases (15%) than *p*‐tau217 (31%; χ2(1)=43.26, 95%CI=‐0.2 – ‐0.11, *p* = 4.8e‐11) and Aβ42/Aβ40 (55%; χ2(1)=220.31, 95%CI=‐0.44 – ‐0.34, *p* = 7.7e‐50).

**Conclusion:**

In this real‐world dataset, we identified effects of comorbidities (LDL‐cholesterol, diabetes, liver, and kidney functioning) on plasma biomarkers. The *p*‐tau217/Aβ42 ratio had low rates of undetermined, and it may help to account for effects of morbidities on plasma levels; still, creatinine remained a significant confound.